# An exploratory microarray analysis of estrogen-mediated gene expression in central pathways that control energy balance in female rats (*Rattus norvegicus*)

**DOI:** 10.1186/s13104-026-07672-2

**Published:** 2026-01-30

**Authors:** Henry Lang, Kaitlin Burch, Dusti Sloan

**Affiliations:** 1 College of Osteopathic Medicine, Oklahoma State University Center for Health Sciences, Tulsa , OK USA; 2 Department of Pharmacology and Physiology, Center for Health Sciences, Oklahoma State University, Tulsa, OK USA

**Keywords:** Arcuate nucleus, Paraventricular nucleus, Nucleus of the solitary tract, Melanocortin signaling, Corticotropin-releasing hormone, Thyrotropin-releasing hormone, Vasopressin, Oxytocin, Bioinformatics

## Abstract

**Objective:**

Obesity and associated conditions are concerning, especially during menopause. Estrogens influence energy balance in key brain regions including the arcuate nucleus (ARC), paraventricular nucleus (PVN), and the nucleus of the solitary tract (NTS). The mechanisms by which estrogens influence energy balance are incompletely understood. Accordingly, we used a microarray analysis to investigate estradiol benzoate (EB)-regulated gene expression within the ARC, PVN, and NTS of ovariectomized rats treated with EB or Oil vehicle. DAVID Bioinformatics was used to identify enriched biological themes.

**Results:**

Within the ARC, EB did not alter gene expression of neuropeptides or receptors known to influence energy balance. In the PVN, EB treatment increased Npy1r and Avp expression, with notable effect sizes in the expression of Oxt, Crh, Trh, and Mc3r. Gene expression in the NTS was minimally affected by EB. Within the PVN, DAVID Bioinformatics revealed enrichment in pathways related to cell signaling. Although variability was high due to small sample size and technical challenges inherent to microdissection, this exploratory study provides preliminary gene expression profiles of EB-regulation within the ARC, PVN, and NTS. These findings may serve as a basis for future, targeted investigations of EB regulation within intracellular signaling pathways.

**Supplementary Information:**

The online version contains supplementary material available at 10.1186/s13104-026-07672-2.

## Introduction

Overweight or obese body habitus predisposes patients to health risks, including cancer, cardiovascular disease, and diabetes [1]. Older women are particularly susceptible to weight gain as they approach menopause [2, 3], and early animal studies confirm that estrogens decrease food intake and body weight [4–7]. Although the association between estrogen deficiency and weight gain is well established [8], the central mechanisms by which estrogens regulate energy intake and expenditure are complex and incompletely understood.

Eating is a complex behavior that is integrated across several brain regions. Communication between these areas involves numerous neurohormones, neurotransmitters, receptors, and intracellular signaling pathways. The arcuate nucleus of the hypothalamus (ARC) responds to nutrients and circulating hormones and communicates with the lateral hypothalamus (LH) to initiate eating, and to the paraventricular nucleus (PVN) of the hypothalamus to inhibit eating. The ARC, LH, and PVN are interconnected and communicate with the hindbrain, including the nucleus of the solitary tract (NTS) [9–11]. Estrogen receptors are located in CNS regions that influence food intake [12–14], and the effect of estrogens on feeding and energy use occur via these receptors [15–20].

Many studies involving gene expression focus on a limited number of genes. DNA microarray technology offers a broad approach to identifying genes that are differentially regulated in response to treatment. We utilized microarray analysis to explore gene expression changes induced by estradiol benzoate (EB). mRNA was isolated from the ARC, PVN, and NTS of ovariectomized (OVX) rats treated with EB (*n* = 3) or Oil vehicle (*n* = 3). We explored genes associated with energy balance and candidate genes for future studies. Microarray results for over 23,000 genes are available in NIH’s Gene Expression Omnibus (GEO).

## Materials and methods

### Surgery

Adult (90 days old), female Sprague-Dawley rats (Charles River 001CD) were bilaterally OVX using a protocol well established in this laboratory [21–23]. Briefly, rats received oral meloxicam (0.15 mL at 1.5 mg/mL), then were anesthetized with isoflurane (3–5% induction; 1.5-3% maintenance). Using a ventral approach, ovaries were removed, and rats were allowed to recover one week before implementing the EB treatment protocol. Body weight was recorded throughout the study to assess animal health and confirm the effects of treatment.

### EB treatment protocol

Animals received subcutaneous injections of either 10 µg EB in 0.1 mL of oil vehicle (EB; *n* = 3) or 0.1 mL of oil vehicle (Oil; *n* = 3) for two consecutive days (see timeline in Additional file 1). This schedule simulates the rise and peak of EB during the proestrus phase of the four-day estrous cycle. Tissues were collected 48 h after the second EB injection, a time point corresponding to maximal physiological and behavioral effects of EB [23]. Uteri were collected and weighed to confirm estrogenic activity [22, 23]. This well-established protocol, adapted from Woolley and McEwen, along with confirmation of EB effects on uterine hypertrophy by Miura et al. eliminates the need to measure circulating EB levels [22–26].

### Tissue collection

On the day of termination, rats were anesthetized with CO_2_ inhalation and decapitated. Following established protocol [25], brains were rapidly extracted, flash-frozen in liquid nitrogen, and stored at -80 °C until tissue punches were collected. Bilateral punches from coronal sections were obtained from the ARC, PVN, and NTS using a 1 × 3 mm tissue punch. Locations were determined using stereotaxic coordinates from the Paxinos and Watson atlas [27], with major landmarks described in previous publications from this lab [28, 29]. Using the following coordinates, samples were collected from the ARC (− 2.64 to − 3.24 from bregma), PVN (− 1.32 to − 1.92 from bregma) and NTS (− 13.56 to − 14.40 from bregma). Tissue was stored in RNAlater and frozen (Ambion, AM7020) until mRNA isolation [25].

### RNA isolation and microarray analysis

RNA was isolated using a Biorad Aurum Total RNA Mini kit (732–6820) per manufacturer’s instructions. RNA was sent to Thermo Fisher Scientific, Inc. (TFS) for transcriptome analysis. The mRNA samples passed all quality control checks. The microarray, Rat Clariom S Pico Assay (Applied Biosystems, 902937), compared gene expression for EB-treated versus Oil-treated rats for over 23,000 genes. TFS required a minimum of three samples per treatment group for the microarray; three samples from the PVN, ARC, and DVC of EB- and Oil-treated rats were provided. Although microarray studies often use fewer replicates due to cost and time, a minimum of three replicates is recommended [30]. Gene notation follows the National Center for Biotechnology Database: proteins are capitalized (e.g., “OXT”), whereas mRNA is referred to as “Oxt”.

### Statistical analysis

Cell intensity (CEL) files from the microarray analysis were background-adjusted, normalized, and log [2] transformed by TFS following methods described by Irizarry [31]. Processed output chip files (CHP) were analyzed using the Transcriptome Analysis Console (TAC) software 4.0. TAC utilizes an empirical Bayes (eBayes) method with one-way ANOVA (t-test not available in this software) to assess statistical significance at *p* < 0.05 and an absolute fold change ≥ 2. The eBayes method is commonly used as a data reduction tool to stabilize variance in studies that include thousands of genes [32]. TAC reports both unadjusted *p*-values and values for false discovery rates (FDR). Data are reported as means ± standard deviation (SD) of signal intensity (Log 2) and fold change (Log 2), with fold change presented as absolute values.

### Overview of exploratory approach

Microarray analyses frequently rely on small sample sizes [30], and this study is no exception. Moreover, micro-dissected regions, like those sampled in our study, exhibit high biological variability due to technical differences in tissue collection. Given these limitations, results should be considered exploratory.

Analyses included a targeted examination of preselected genes involved in melanocortin signaling, as well as a transcriptome-wide survey of differentially expressed genes (DEGs) with potential biological relevance. Genes associated with melanocortin signaling and downstream effects were investigated as “genes of interest” in the ARC, PVN, and NTS. To control for type I error, FDR was initially calculated by TAC across all 23,188 genes. For preselected gene subsets, *q*-values were calculated using the Benjamini-Hochberg procedure (Excel). Effect sizes were calculated using Cohen’s d equation (Excel).

For the transcriptome-wide survey of EB-regulated genes, DEGs identified by TAC with unadjusted *p*-values ≤ 0.05 and fold changes ≥ │2│ were selected for further analysis. These DEGs were visualized using heatmaps and analyzed using DAVID (Database for Annotation, Visualization, and Integrated Discovery), an NIH bioinformatics tool that organizes large datasets into meaningful biological categories [33, 34]. Genes from each brain region were sorted by up- or down-regulation by EB. DAVID functional annotation clustering results are reported using three Gene Ontology (GO) categories that, with some overlap, represent groups of genes involved in biological processes, cellular components, and molecular function: GOTERM_BP_FAT (BP = Biological Process), GOTERM_CC_FAT (CC = Cellular Component), and GOTERM_MF_FAT (MF = Molecular Function).

## Results

All microarray data, including CEL files and CHP files, have been deposited in NCBI GEO under accession number GSE293502. Biological activity of EB was confirmed by measuring uterine weights [22, 23, 26]. An independent t-test revealed that uterine weights in EB-treated animals were significantly higher than in controls [*t*(4) = -13.07, *p* < 0.001].

### ARC, PVN, NTS heatmaps—genes of interest

Heatmaps of selected genes of interest within the ARC, PVN, and NTS (Additional file 2) show that EB-treated and Oil-treated samples did not form distinct clusters, indicating substantial within-group variability.

### ARC—genes of interest

**Fig. 1:**
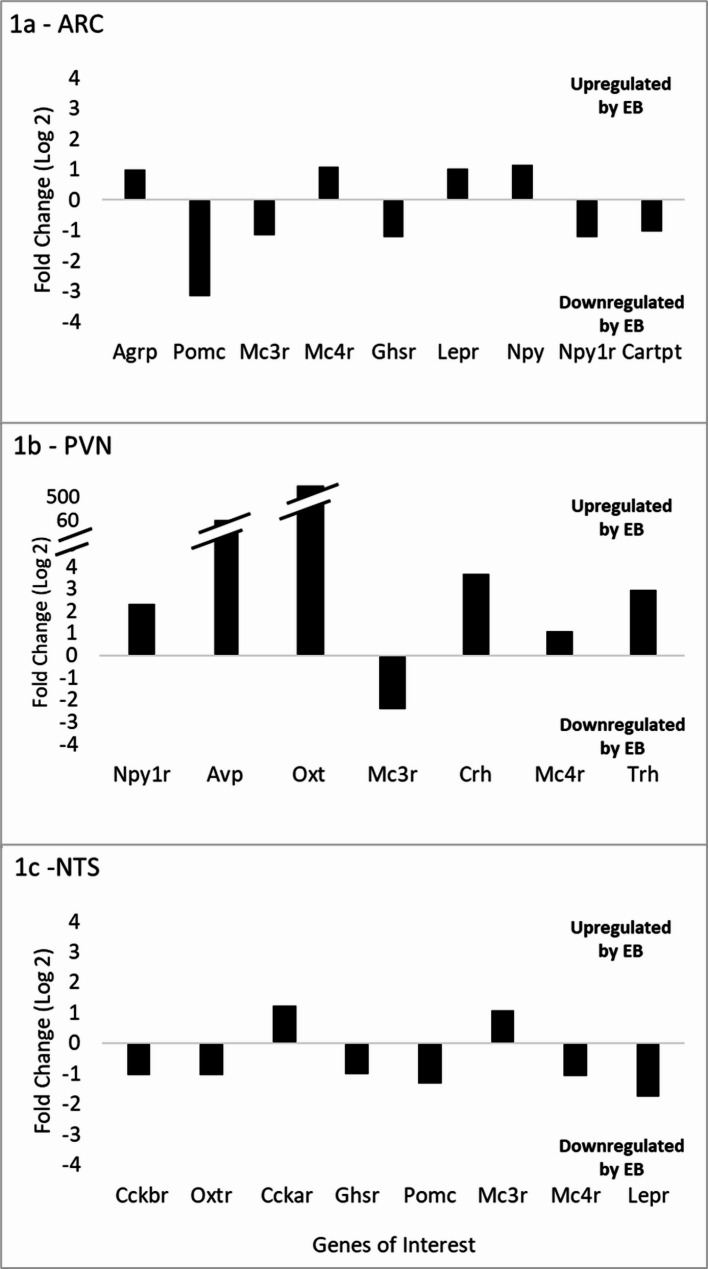
Fold change differences in gene expression for EB- vs. Oil-treated rats in the ARC (1a), PVN (1b), and NTS (1c). Positive values indicate upregulation by EB; negative values indicate downregulation. Each bar represents the fold change for genes of interest

EB did not significantly affect the expression of receptors for ghrelin (Ghsr), leptin (Lepr), melanocortin 3 (Mc3r) or 4 (Mc4r), or neuropeptide Y (Npy1r). In addition, EB did not cause significant differential expression of neuropeptide Y (Npy), agouti-related peptide (AgRP), CART pre-propeptide (Cartpt), or proopiomelanocortin (Pomc), although Pomc, the precursor to α-MSH, was threefold higher in the oil-treated group (fold change = 3.17) with a medium effect size (*d* = 0.52) (Fig. 1a, Table 1a).

### PVN—genes of interest

In terms of receptors in the PVN, EB did not significantly affect the expression of Mc4r or Mc3r after correction for multiple testing. However, Mc3r expression was twofold higher in the Oil-treated group with a large effect size (Fold change = 2.44, *d* = 1.64). Npy1r expression was significantly higher in the EB-treated group with a notable fold change and effect size (Fold change = 2.31; *q* = 0.001; *d* = 5.7). Vasopressin (Avp) expression was significantly higher in the EB-treated group with a notable fold change and effect size (Fold change = 57.11, *q* = 0.025, *d* = 2.71). Although not statistically significant, oxytocin (Oxt) expression showed a large fold change and effect size, suggesting potential biological relevance (Fold change = 506.15, *q* = 0.098, *d* = 2.53). Corticotropin-releasing hormone (Crh) and thyrotropin-releasing hormone (Trh) expression levels were approximately three-fold higher in the EB-treated group, though not statistically significant (Crh: Fold change = 3.66, *q* = 0.146, *d* = 1.73; Trh: Fold change = 2.97, *q* = 0.300, *d* = 1.19) (Fig. 1b, Table 1b).

### NTS—genes of interest

Within the NTS, EB did not significantly affect the expression of receptors for cholecystokinin A (Cckar) or B (Cckbr), oxytocin (Oxtr), Ghsr, Mc3r, Mc4r, and Lepr, and did not induce differential expression of Pomc (Fig. 1c, Table 1c).

**Table 1:** Analyses for genes of interest in the ARC, PVN, and NTS

Gene (Symbol)	Public Gene ID RefSeq mRNA	Oil Signal Intensity Log 2 (SD)	EB Signal Intensity Log 2 (SD)	Fold Change Log 2	Unadjusted p-value	Benjamini-Hochberg Adjusted *p*-value (q)	Cohen’s d Effect size (d)
**1a-ARC Genes of Interest**							
Agouti related neuropeptide (Agrp)	NM_033650	6.84 (1.57)	6.86 (0.91)	1.01	0.299	0.858	0.02
Proopiomelanocortin (Pomc)	NM_139326	11.5 (1.98)	9.84 (4.01)	**– 3.17**	0.337	0.858	**0.52**
Melanocortin 3 receptor (Mc3r)	NM_001025270	8.73 (1.01)	8.51 (0.91)	– 1.16	0.389	0.858	0.23
Melanocortin 4 receptor (Mc4r)	NM_013099	6.9 (0.06)	7.03 (0.11)	1.09	0.452	0.858	**1.47**
Ghrelin receptor (Ghsr)	NM_032075	9.63 (0.28)	9.32 (0.21)	– 1.23	0.562	0.858	**1.25**
Leptin receptor (Lepr)	NM_012596	9.65 (0.09)	9.7 (0.63)	1.03	0.585	0.858	0.11
Neuropeptide Y (Npy)	NM_012614	13.63 (0.92)	13.44 (0.68)	1.14	0.667	0.858	0.24
Neuropeptide Y receptor 1 (Npy1r)	NM_001113357	9.99 (0.24)	9.7 (0.79)	– 1.22	0.796	0.870	**0.50**
CART prepropeptide (Cartpt)	NM_017110	15.2 (0.76)	15.12 (0.75)	– 1.05	0.870	0.870	0.11
**1b-PVN Genes of Interest**							
Neuropeptide Y receptor 1 (Npy1r)	NM_001113357	8.73 (0.01)	9.94 (0.3)	**2.31**	0.0002	**0.001**	**5.7**
Arginine vasopressin (Avp)	NM_016992	6.96 (2.03)	12.79 (2.27)	**57.11**	0.007	**0.025**	**2.71**
Oxytocin (Oxt)	NM_012996	6.95 (4.57)	15.93 (2.07)	**506.15**	0.042	0.098	**2.53**
Melanocortin 3 receptor (Mc3r)	NM_001025270	8.32 (0.69)	7.03 (0.87)	**2.44**	0.103	0.146	**1.64**
Corticotropin-releasing hormone (Crh)	NM_031019	7.5 (0.93)	9.37 (1.21)	**3.66**	0.104	0.146	**1.73**
Melanocortin 4 receptor (Mc4r)	NM_013099	7.95 (0.21)	8.09 (0.6)	1.1	0.153	0.178	0.31
Thyrotropin-releasing hormone (Trh)	NM_013046	5.36 (0.63)	6.93 (1.75)	**2.97**	0.300	0.300	**1.19**
**1c-NTS Genes of Interest**							
Cholesytokinin B (Cckbr)	NM_013165	7.22 (0.32)	7.16 (0.18)	1.04	0.115	0.917	0.23
Oxytocin receptor (Oxtr)	NM_012871	5.15 (0.08)	5.08 (0.27)	1.05	0.355	0.927	0.35
Cholecystokinin A (Cckar)	NM_012688	7.26 (0.56)	7.56 (0.61)	1.24	0.496	0.927	0.51
Growth hormone secretagogue receptor (Ghsr)	NM_032075	8.65 (0.07)	8.61 (0.15)	1.02	0.571	0.927	0.34
Proopiomelanocortin (Pomc)	NM_139326	5.22 (0.5)	4.8 (0.36)	1.33	0.797	0.927	0.96
Melanocortin 3 receptor (Mc3r)	NM_001025270	4.64 (0.12)	4.76 (0.13)	1.09	0.825	0.927	0.95
Melanocortin 4 receptor (Mc4r)	NM_013099	8.36 (0.23)	8.25 (0.11)	1.08	0.866	0.927	0.61
Leptin Receptor (Lepr)	NM_012596	8.64 (0.16)	7.83 (1.43)	1.75	0.927	0.927	0.79

Within the ARC (a), PVN (b), and NTS (c), genes, symbols and Public Gene IDs are listed. Data generated using TAC include mean signal intensities (log 2), standard deviation (SD), absolute fold change (log 2), and unadjusted p-values. Benjamini-Hochberg adjusted q-values were calculated for selected genes and Cohen’s d equation was used to calculate effect sizes (d).

Bold text delineates the genes investigated within each brain region (ARC, PVN, and NTS)

### ARC, PVN, NTS heatmaps—transcriptome-wide survey of DEGs.

Heatmaps representing transcriptome-wide differentially expressed genes are included in Additional file 3. Within the ARC and NTS, EB- and Oil-treated samples formed distinct clusters. DEGs within the PVN showed that EB-treated samples clustered together, but Oil-treated sample, 9SR4406A_B03, did not cluster with the other two oil-treated samples.

### ARC—transcriptome-wide survey of DEGs and DAVID bioinformatics

TAC software identified 24 genes upregulated and 75 genes downregulated by EB, and a gene list is included in Additional file 4, tab a.

GO analysis of upregulated genes, conducted using DAVID revealed no statistically significant terms within the categories GOTERM_BP_FAT or GOTERM_CC_FAT after Benjamini-Hochberg correction. Within the GOTERM_MF_FAT category, “cation binding” was significantly enriched (GO: 0043169, fold-enrichment = 2.75, *q* = 0.04). Genes contributing to this enrichment included RET, S100G, CHRNB3, TRPC3, CD93, EBF1, KCNA4, EBF3, RXFP1, SLC6A3, CALB2, NR4A2, ATP13A5, and TRIM16 suggesting that EB may be associated with molecular pathways related to cation binding. Downregulated genes were not significantly enriched under GOTERM_MF_FAT. However, there were many genes associated with GOTERM_BP_FAT and GOTERM_CC_FAT, and most of these genes were associated with reproductive processes (e.g., germ cell development and motility).

### PVN—transcriptome-wide survey of DEGs and DAVID bioinformatics

TAC software identified 51 genes upregulated and 50 genes downregulated by EB and data for these genes are included in Additional file 4, tab b.

GO analysis of upregulated genes (Additional file 5, tab a), showed significant enrichment within all three major categories. Within GOTERM_BP_FAT, four groups were significantly enriched for regulation of biological quality, cell communication, neuropeptide signaling, and signaling. GO GOTERM_CC_FAT showed significant enrichment for cell junction, dense core granule, synapse, neuronal dense core vesicle, and secretory vesicle. Lastly, within GOTERM_MF_FAT, two groups were significantly enriched for neuropeptide receptor activity and neuropeptide Y receptor activity.

GO analysis of downregulated genes (Additional file 5, tab b) showed significant enrichment within the biological process and cellular component categories, but not in the molecular function category. Within enriched categories many groups are clearly associated with cell signaling.

### NTS—transcriptome-wide survey of DEGs and DAVID bioinformatics.

TAC software identified 13 genes upregulated and 8 downregulated by EB treatment (Additional file 4, tab c). Only one gene, ankyrin repeat and SOCS box containing 15 (Asb15), met the significance threshold after FDR correction.

DAVID analysis of upregulated and downregulated genes showed no significant enrichment in either of the three GO categories.

## Discussion

This study examined the effects of EB on gene expression in the ARC, PVN, and NTS, three brain regions involved in energy balance [12–14].

In the ARC, EB did not significantly alter Pomc expression, although a threefold decrease was observed with a medium effect size. Expression of key neuropeptides and their receptors in the ARC remained unchanged following EB treatment. This trend suggests possible physiological feedback suppression due to prolonged EB exposure (i.e., samples were collected 48 h after the second EB injection instead of at an earlier timepoint) [35–38]. GO analysis indicated enrichment in genes associated with cation binding, particularly those involved in ion transport, neurotransmission, and other intracellular signaling pathways. For instance, TRPC3 which was up-regulated by EB (Fold change = 2.7; unadjusted *p*-value = 0.02), is involved in calcium signaling [39]. While calcium is a well-established second messenger, its specific role in energy balance within the ARC remains unclear.

In the PVN, Npy1r expression was significantly increased in EB-treated animals, with a large effect size. Npy1r expression promotes feeding when it binds NPY in the LH. In the ARC, NPY inhibits POMC/CART neurons from activating the satiety center [10, 11]. The upregulation of Npy1r seems contradictory to the known anorexigenic effects of EB, and body weight was reduced, as expected based on previous work, in the EB-treated group of our study. This result may reflect time- and/or dose-dependent regulatory mechanisms. Though not statistically significant, expression of Oxt, Crh, and Trh was higher in the EB-treated group, these hormones are associated with decreased food intake and body weight. EB also increased expression of Avp, a gene of growing interest in satiety research [40–42]. GO analysis of PVN genes suggested that EB influences cell communication, for example by increasing expression of hormones and receptors that are involved in energy homeostasis (i.e. Oxt, Avp, Npy1r).

In the NTS, no significant EB-mediated changes were observed in Pomc, melanocortin receptors, Oxtr, Lepr, Ghsr, or cholecystokinin receptors. Analysis of differentially expressed genes revealed that EB upregulates Asb15. This gene is known to regulate skeletal muscle growth and differentiation in rodent models, mostly through modulation of signal transduction pathways [43, 44]. To our knowledge, this is the first report of Asb15 regulation by EB in the NTS or elsewhere. However, beyond the assumption that EB directly or indirectly modulates Asb15 transcription, the significance of this finding remains unclear given the current literature. GO analysis of genes regulated in the NTS did not reveal significant enrichment in any of the functional categories.

## Limitations

Our sample size was limited (*n* = 3 per group). In addition, technical challenges associated with microdissection can lead to increased variability, and our data clearly indicated variability within groups. Furthermore, the use of pharmacological doses of exogenous EB, as employed in this study, presents inherent limitations (e.g., decreased receptor sensitivity). Our study was also restricted to a single time point limiting any temporal findings. Future studies using physiological doses of EB, with tissue sampling at multiple time points, may clarify our understanding of feedback mechanisms involved in these pathways. Finally, surgical menopause causes immediate effects on food intake and body weight [22, 23, 25]. Though our lab is interested in the immediate changes, it should be noted that many other effects of menopause may take several weeks or longer [45], and these long-term changes are not represented in this data set. Given the limitations of this study, results should be interpreted with caution and used to design future experiments with an increased sample size.

## Supplementary Information

Below is the link to the electronic supplementary material.

Additional file 1. Timeline of Events. A timeline illustrating the sequence of experimental procedures is provided, beginning with arrival of rats at the facility, followed by ovariectomy, implementation of hormone treatment, termination and tissue collection.

Additional file 2. Heatmaps_Selected Genes. Heatmaps for genes of interest in the ARC, PVN, and NTS from EB- vs. Oil-treated samples. Heatmaps were generated using TAC to visualize the expression of selected genes of interest in the ARC (a), PVN (b), and NTS (c). Each column represents an individual sample; each row represents a single gene. Expression values are shown as log 2 transformed signal intensities. Red indicates the highest expression and blue indicates the lowest

Additional file 3. Heatmaps_DEGs. Differentially expressed genes (DEGs) in the ARC, PVN, and NTS from EB-vs. Oil-treated samples. Heatmaps were generated using TAC to visualize DEGs in the ARC (a), PVN (b), NTS (c). Genes were selected based on unadjusted *p*-values ≤ 0.05 and absolute fold change > 2. With the exception of Asb15 in the NTS, no genes met significance thresholds after FDR correction, therefore these heatmaps are presented for exploratory purposes only. Each column represents an individual sample, and each row represents a single gene. Expression values are shown as log 2 transformed signal intensities. Red indicates the highest expression, and blue indicates the lowest.

Additional file 4. Transcriptome-wide DEGs. Transcriptome-wide DEGs within the ARC (tab a), PVN, (tab b) and NTS (tab c). This file contains lists of DEGs (EB- vs. Oil-treated) in the ARC, PVN, and NTS generated by TAC. No genes met significance thresholds after Benjamini-Hochberg correction, except Asb15 in the NTS. These data are provided for transparency and to support exploratory analysis using the DAVID Bioinformatics Tool.

Additional file 5. GO Analysis of EB-upregulated and downregulated genes in the PVN. EB-upregulated genes in the PVN were analyzed using the DAVID Bioinformatics Tool (tab a). Significant enrichment was observed across all three GO categories. GO terms and associated genes are listed along with fold enrichment values and Benjamini-Hochberg adjusted *q*-values. EB-downregulated genes (tab b) showed significant enrichment in two categories: biological process and cellular component. GO terms and associated genes are reported with fold enrichment values and Benjamini-Hochberg adjusted *q*-values.

## Data Availability

All microarray data, including CEL files and CHP files, have been deposited in the NCBI Gene Expression Omnibus (GEO) at [https://www.ncbi.nlm.nih.gov/geo/]: Sloan D, Burch K, Lang H. (2025). Estrogen-mediated gene expression in the arcuate (ARC) and paraventricular (PVN) nuclei of the hypothalamus and the nucleus of the solitary tract (NTS) in female rats (Rattus norvegicus). Gene Expression Omnibus GSE293502.

## Abbreviations

ARC: Arcuate nucleus of the hypothalamus
CEL: Cell intensity files
CHP: Processed output chip files
EB: Estradiol benzoate
NTS: Nucleus of the solitary tract
OVX: Ovariectomized
PVN: Paraventricular nucleus of the hypothalamus
TAC: Transcriptome Analysis Console
TFS: Thermo Fisher Scientific, Inc.

## References

[1] Blüher M. Obesity: global epidemiology and pathogenesis. Nat Rev Endocrinol. 2019;15(5):288–98. 30814686 10.1038/s41574-019-0176-8

[2] Greendale GA, Sternfeld B, Huang M, Han W, Karvonen-Gutierrez C, Ruppert K, et al. Changes in body composition and weight during the menopause transition. JCI Insight. 2019;4(5):e124865. 30843880 10.1172/jci.insight.124865 PMC6483504

[3] Marlatt KL, Pitynski-Miller DR, Gavin KM, Moreau KL, Melanson EL, Santoro N, et al. Body composition and cardiometabolic health across the menopause transition. Obes (Silver Spring). 2022;30(1):14–27. 10.1002/oby.23289 PMC8972960 34932890

[4] Blaustein JD, Wade GN. Ovarian influences on the meal patterns of female rats. Physiol Behav. 1976;17(2):201–8. 1033580 10.1016/0031-9384(76)90064-0

[5] Drewett RF. The meal patterns of the oestrous cycle and their motivational significance. Q J Exp Psychol. 1974;26(Pt3):489–94. 4472372 10.1080/14640747408400438

[6] Tarttelin MF, Gorski RA. Variations in food and water intake in the normal and acyclic female rat. Physiol Behav. 1971;7(6):847–52. 5167385 10.1016/0031-9384(71)90050-3

[7] Wade GN, Gray JM. Gonadal effects on food intake and adiposity: a metabolic hypothesis. Physiol Behav. 1979;22(3):583–93. 379889 10.1016/0031-9384(79)90028-3

[8] Asarian L, Geary N. Sex differences in the physiology of eating. Am J Physiol Regul Integr Comp Physiol. 2013;305(11):R1215–1267. 23904103 10.1152/ajpregu.00446.2012 PMC3882560

[9] Alcantara IC, Tapia APM, Aponte Y, Krashes MJ. Acts of appetite: neural circuits governing the appetitive, consummatory, and terminating phases of feeding. Nat Metab. 2022;4(7):836–47. 35879462 10.1038/s42255-022-00611-y PMC10852214

[10] Morton GJ, Cummings DE, Baskin DG, Barsh GS, Schwartz MW. Central nervous system control of food intake and body weight. Nature. 2006;443(7109):289–95. 16988703 10.1038/nature05026

[11] Schwartz MW, Woods SC, Porte D, Seeley RJ, Baskin DG. Central nervous system control of food intake. Nat 2000. 2000;404(6778):6778. 10.1038/35007534 10766253

[12] Laflamme N, Nappi RE, Drolet G, Labrie C, Rivest S. Expression and neuropeptidergic characterization of Estrogen receptors (ERalpha and ERbeta) throughout the rat brain: anatomical evidence of distinct roles of each subtype. J Neurobiol. 1998;36(3):357–78. 9733072 10.1002/(sici)1097-4695(19980905)36:3<357::aid-neu5>3.0.co;2-v

[13] Osterlund M, Kuiper GG, Gustafsson JA, Hurd YL. Differential distribution and regulation of Estrogen receptor-alpha and -beta mRNA within the female rat brain. Brain Res Mol Brain Res. 1998;54(1):175–80. 9526077 10.1016/s0169-328x(97)00351-3

[14] Shughrue PJ, Dellovade TL, Merchenthaler I. Estrogen modulates Oxytocin gene expression in regions of the rat supraoptic and paraventricular nuclei that contain Estrogen receptor-beta. Prog Brain Res. 2002;139:15–29. 12436923 10.1016/s0079-6123(02)39004-6

[15] Geary N, Asarian L, Korach KS, Pfaff DW, Ogawa S. Deficits in E2-dependent control of feeding, weight gain, and cholecystokinin satiation in ER-alpha null mice. Endocrinology. 2001;142(11):4751–7. 11606440 10.1210/endo.142.11.8504

[16] Heine PA, Taylor JA, Iwamoto GA, Lubahn DB, Cooke PS. Increased adipose tissue in male and female Estrogen receptor-alpha knockout mice. Proc Natl Acad Sci U S A. 2000;97(23):12729–34. 11070086 10.1073/pnas.97.23.12729 PMC18832

[17] Roesch DM. Effects of selective Estrogen receptor agonists on food intake and body weight gain in rats. Physiol Behav. 2006;87(1):39–44. 16181647 10.1016/j.physbeh.2005.08.035

[18] Santollo J, Katzenellenbogen BS, Katzenellenbogen JA, Eckel LA. Activation of ERα is necessary for estradiol’s anorexigenic effect in female rats. Horm Behav. 2010;58(5):872–7. 20807534 10.1016/j.yhbeh.2010.08.012 PMC2982904

[19] Santollo J, Eckel LA. Effect of a putative ERalpha antagonist, MPP, on food intake in cycling and ovariectomized rats. Physiol Behav. 2009;97(2):193–8. 19254732 10.1016/j.physbeh.2009.02.021 PMC2699763

[20] Thammacharoen S, Geary N, Lutz TA, Ogawa S, Asarian L. Divergent effects of estradiol and the Estrogen receptor-alpha agonist PPT on eating and activation of PVN CRH neurons in ovariectomized rats and mice. Brain Res. 2009;1268:88–96. 19281799 10.1016/j.brainres.2009.02.067

[21] Askew ML, Muckelrath HD, Johnston JR, Curtis KS. Neuroanatomical association of hypothalamic HSD2-containing neurons with ERα, catecholamines, or oxytocin: implications for feeding? Front Syst Neurosci. 2015;9:91. 26124709 10.3389/fnsys.2015.00091 PMC4466453

[22] Curtis KS. Estradiol, and osmolality: behavioral responses and central pathwayS. Physiol Behav. 2015;152(0 0):422–30. 10.1016/j.physbeh.2015.06.017 PMC4661113 26074202

[23] Graves NS, Hayes H, Fan L, Curtis KS. Time course of behavioral, physiological, and morphological changes after estradiol treatment of ovariectomized rats. Physiol Behav. 2011;103(3–4):261–7. 21324332 10.1016/j.physbeh.2011.02.017 PMC3476457

[24] Woolley CS, McEwen BS. Estradiol regulates hippocampal dendritic spine density via an N-methyl-D-aspartate receptor-dependent mechanism. J Neurosci. 1994;14(12):7680–7. 7996203 10.1523/JNEUROSCI.14-12-07680.1994 PMC6576901

[25] Sloan DK, Spencer DS, Curtis KS. Estrogen effects on oxytocinergic pathways that regulate food intake. Horm Behav. 2018;105:128–37. 30118729 10.1016/j.yhbeh.2018.08.007

[26] Miura S, Tsong YY, Koide SS. Hormonal effects of estrogen-protein conjugates on rat uterus. Biol Reprod. 1971;5(3):340–2. 4360204 10.1093/biolreprod/5.3.340

[27] Paxinos G, Watson C. The rat brain in stereotaxic coordinates. Academic; 1998. p. 237.

[28] Burch KE, McCracken K, Buck DJ, Davis RL, Sloan DK, Curtis KS. Relationship between Circulating metabolic hormones and their central receptors during Ovariectomy-Induced weight gain in Rats. Front Physiol. 2022;12. 10.3389/fphys.2021.800266 PMC8766843 35069259

[29] Curtis KS, McCracken K, Espinosa E, Ong J, Buck DJ, Davis RL. Temporal and Site-Specific changes in central neuroimmune factors during rapid weight gain after ovariectomy in rats. Neurochem Res. 2018;43(9):1802–13. 30030770 10.1007/s11064-018-2596-6

[30] Lee MLT, Kuo FC, Whitmore GA, Sklar J. Importance of replication in microarray gene expression studies: statistical methods and evidence from repetitive cDNA hybridizations. Proc Natl Acad Sci U S A. 2000;97(18):9834–9. 10963655 10.1073/pnas.97.18.9834 PMC27599

[31] Irizarry RA, Hobbs B, Collin F, Beazer-Barclay YD, Antonellis KJ, Scherf U, et al. Exploration, normalization, and summaries of high density oligonucleotide array probe level data. Biostatistics. 2003;4(2):249–64. 12925520 10.1093/biostatistics/4.2.249

[32] Efron B, Tibshirani R, Storey JD, Tusher V. Empirical Bayes analysis of a microarray experiment. J Am Stat Assoc. 2001;96(456):1151–60.

[33] Huang DW, Sherman BT, Lempicki RA. Systematic and integrative analysis of large gene lists using DAVID bioinformatics resources. Nat Protoc. 2009;4(1):44–57. 19131956 10.1038/nprot.2008.211

[34] Sherman BT, Hao M, Qiu J, Jiao X, Baseler MW, Lane HC, et al. DAVID: a web server for functional enrichment analysis and functional annotation of gene lists (2021 update). Nucleic Acids Res. 2022;50(W1):W216–21. 35325185 10.1093/nar/gkac194 PMC9252805

[35] Rosie R, Thomson E, Blum M, Roberts JL, Fink G. Oestrogen positive feedback reduces arcuate Proopiomelanocortin messenger ribonucleic acid. J Neuroendocrinol. 1992;4(5):625–30. 21554648 10.1111/j.1365-2826.1992.tb00212.x

[36] Petersen SL, Keller ML, Carder SA, McCrone S. Differential effects of Estrogen and progesterone on levels of POMC mRNA levels in the arcuate nucleus: relationship to the timing of LH surge release. J Neuroendocrinol. 1993;5(6):643–8. 8680436 10.1111/j.1365-2826.1993.tb00534.x

[37] Wilcox JN, Roberts JL. Estrogen decreases rat hypothalamic Proopiomelanocortin messenger ribonucleic acid levels. Endocrinology. 1985;117(6):2392–6. 2933246 10.1210/endo-117-6-2392

[38] Pelletier G, Li S, Luu-The V, Labrie F. Oestrogenic regulation of pro-opiomelanocortin, neuropeptide Y and corticotrophin-releasing hormone mRNAs in mouse hypothalamus. J Neuroendocrinol. 2007;19(6):426–31. 17388940 10.1111/j.1365-2826.2007.01548.x

[39] Trebak M, Vazquez G, Bird GSJ, Putney JW. The TRPC3/6/7 subfamily of cation channels. Cell Calcium. 2003;33(5–6):451–61. 12765690 10.1016/s0143-4160(03)00056-3

[40] Pei H, Sutton AK, Burnett KH, Fuller PM, Olson DP. AVP neurons in the paraventricular nucleus of the hypothalamus regulate feeding. Mol Metab. 2014;3(2):209–15. 24634830 10.1016/j.molmet.2013.12.006 PMC3953699

[41] Brot MD, De Vries GJ, Dorsa DM. Local implants of testosterone metabolites regulate vasopressin mRNA in sexually dimorphic nuclei of the rat brain. Peptides. 1993;14(5):933–40. 8284269 10.1016/0196-9781(93)90069-s

[42] Sanada K, Yoshimura M, Ikeda N, Baba K, Nishimura H, Nishimura K, et al. Chemogenetic activation of endogenous arginine vasopressin exerts anorexigenic effects via central nesfatin-1/NucB2 pathway. J Physiol Sci. 2021;71(1):18. 34134629 10.1186/s12576-021-00802-4 PMC10717637

[43] McDaneld TG, Hancock DL, Moody DE. Altered mRNA abundance of ASB15 and four other genes in skeletal muscle following administration of beta-adrenergic receptor agonists. Physiol Genomics. 2004;16(2):275–83. 14645738 10.1152/physiolgenomics.00127.2003

[44] McDaneld TG, Spurlock DM. Ankyrin repeat and suppressor of cytokine signaling (SOCS) box-containing protein (ASB) 15 alters differentiation of mouse C2C12 myoblasts and phosphorylation of mitogen-activated protein kinase and Akt. J Anim Sci. 2008;86(11):2897–902. 18641171 10.2527/jas.2008-1076

[45] Moiety FM, Salem HA, Mehanna RA, Abdel-Ghany BS. Comparative study on induction and effects of surgical menopause in a female rat model: a prospective case control study. Int J Clin Exp Med. 2015;8(6):9403–11. 26309602 PMC4538166

