# Supplementary figures and images for: An exploratory microarray analysis of estrogen-mediated gene expression in central pathways that control energy balance in female rats (Rattus norvegicus)
Source: BMC Res Notes. 2026 Jan 30;19:88. doi: 10.1186/s13104-026-07672-2 (PMC12930718; doi:10.1186/s13104-026-07672-2)

# Timeline of Events

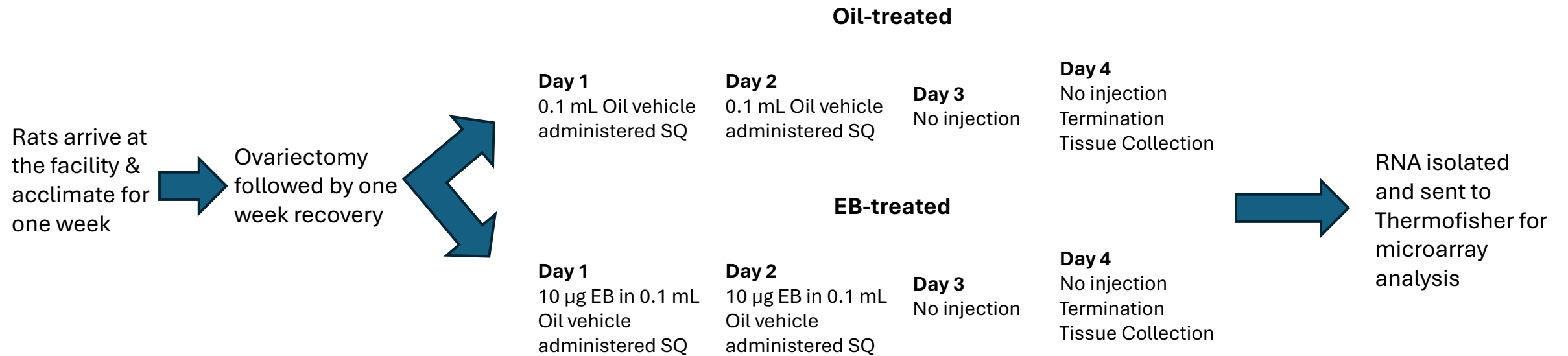

Supplement: Supplementary file 1 — Additional file 1. Timeline of Events. A timeline illustrating the sequence of experimental procedures is provided, beginning with arrival of rats at the facility, followed by ovariectomy, implementation of hormone treatment, termination and tissue collection. [file 13104_2026_7672_MOESM1_ESM.pdf]
