# Supplementary material for: An exploratory microarray analysis of estrogen-mediated gene expression in central pathways that control energy balance in female rats (Rattus norvegicus)
Source: BMC Res Notes. 2026 Jan 30;19:88. doi: 10.1186/s13104-026-07672-2 (PMC12930718; doi:10.1186/s13104-026-07672-2)

Additional File 2_Heatmaps_Selected Genes

Title: Heatmaps for genes of interest in the ARC, PVN, and NTS from EB- vs. Oil-treated samples

Description of Data:

Heatmaps were generated using TAC to visualize the expression of selected genes of interest in the:

- ARC (a)
- PVN (b)
- NTS (c)

Each column represents an individual sample; each row represents a single gene. Expression values are shown as log 2 transformed signal intensities. Red indicates the highest expression and blue indicates the lowest.

2a ARC - Selected genes of interest in EB- vs. Oil-treated samples

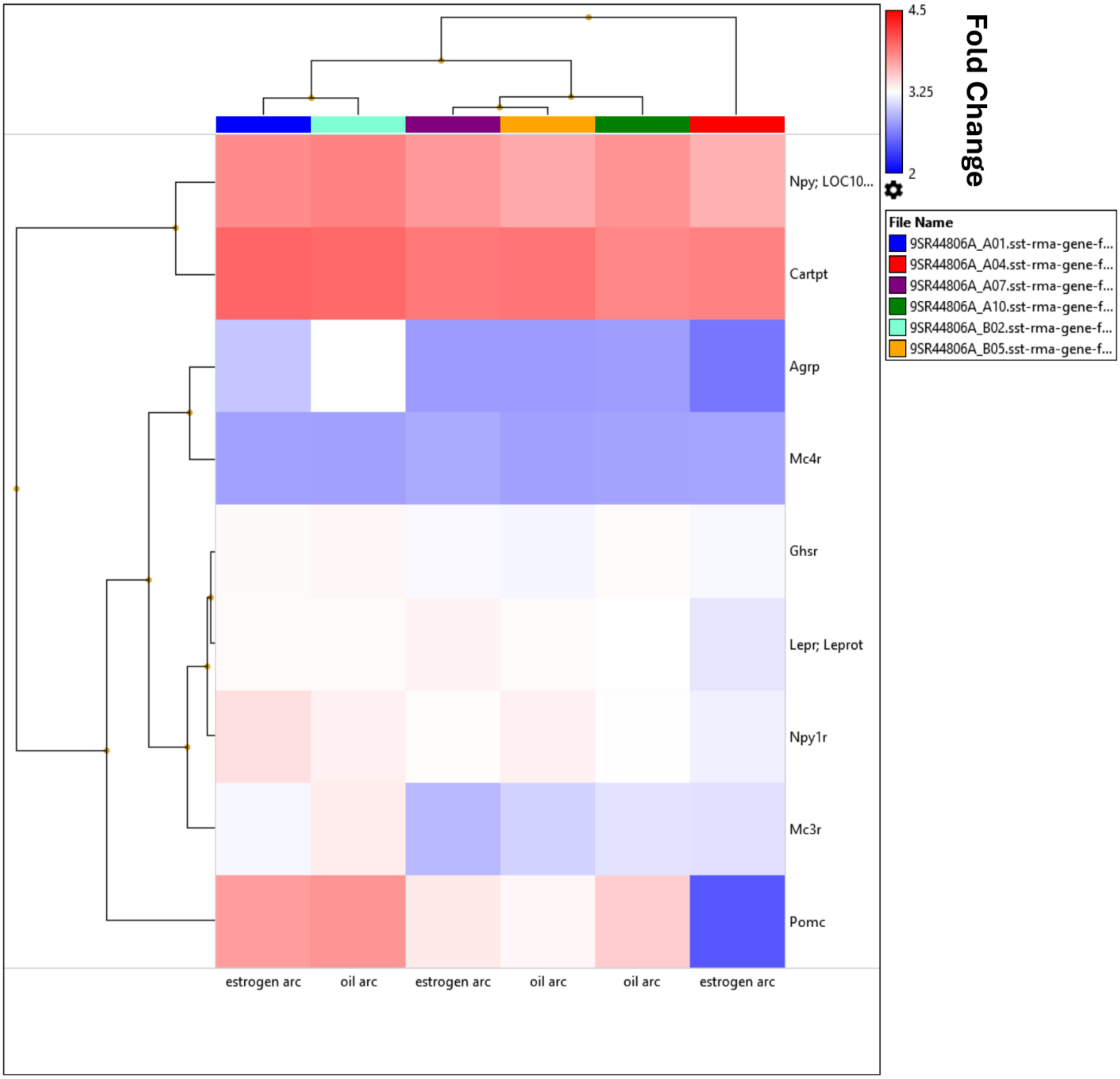

2b PVN - Selected genes of interest in EB- vs. Oil-treated samples

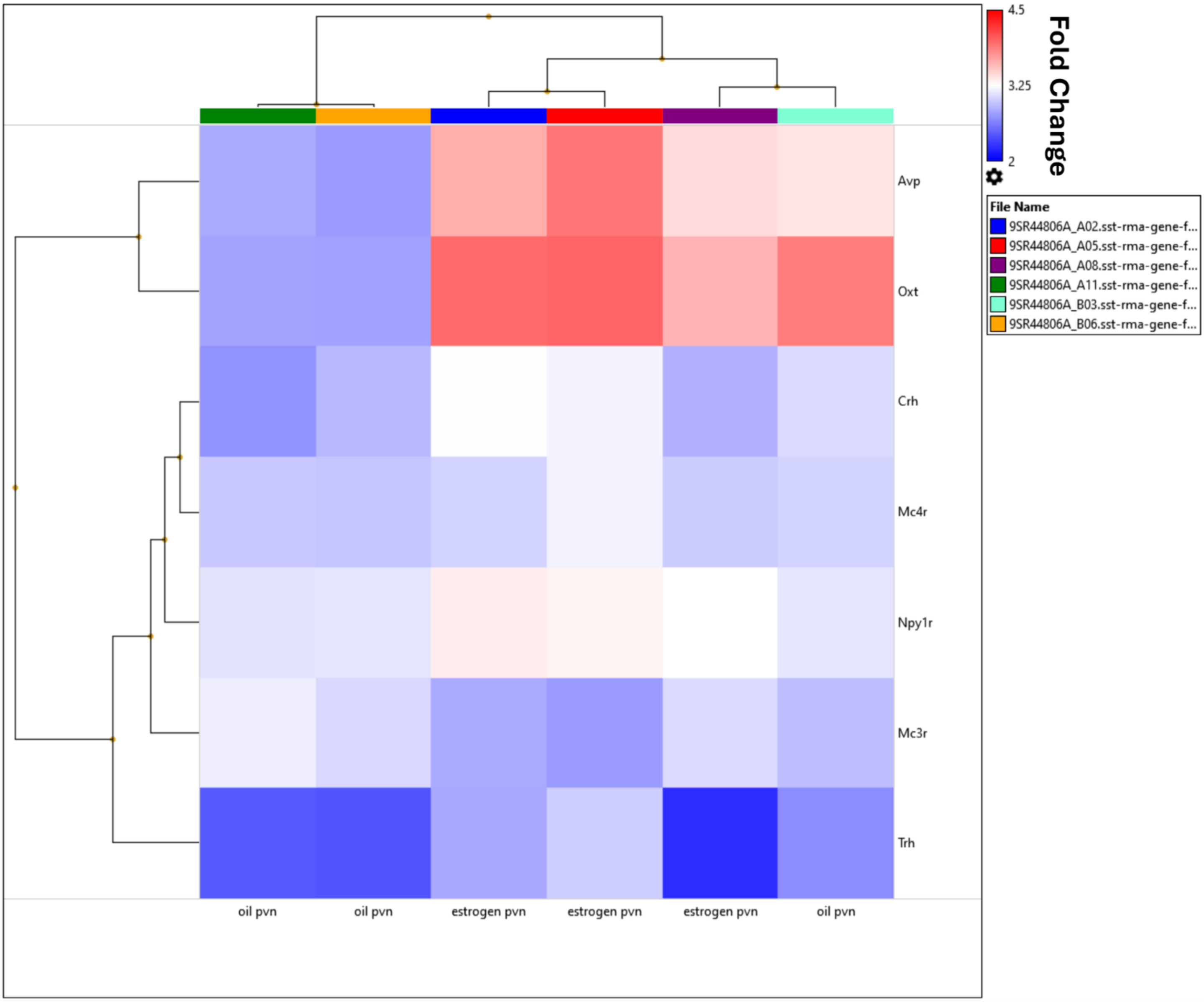

2c NTS - Selected genes of interest in EB- vs. Oil-treated samples

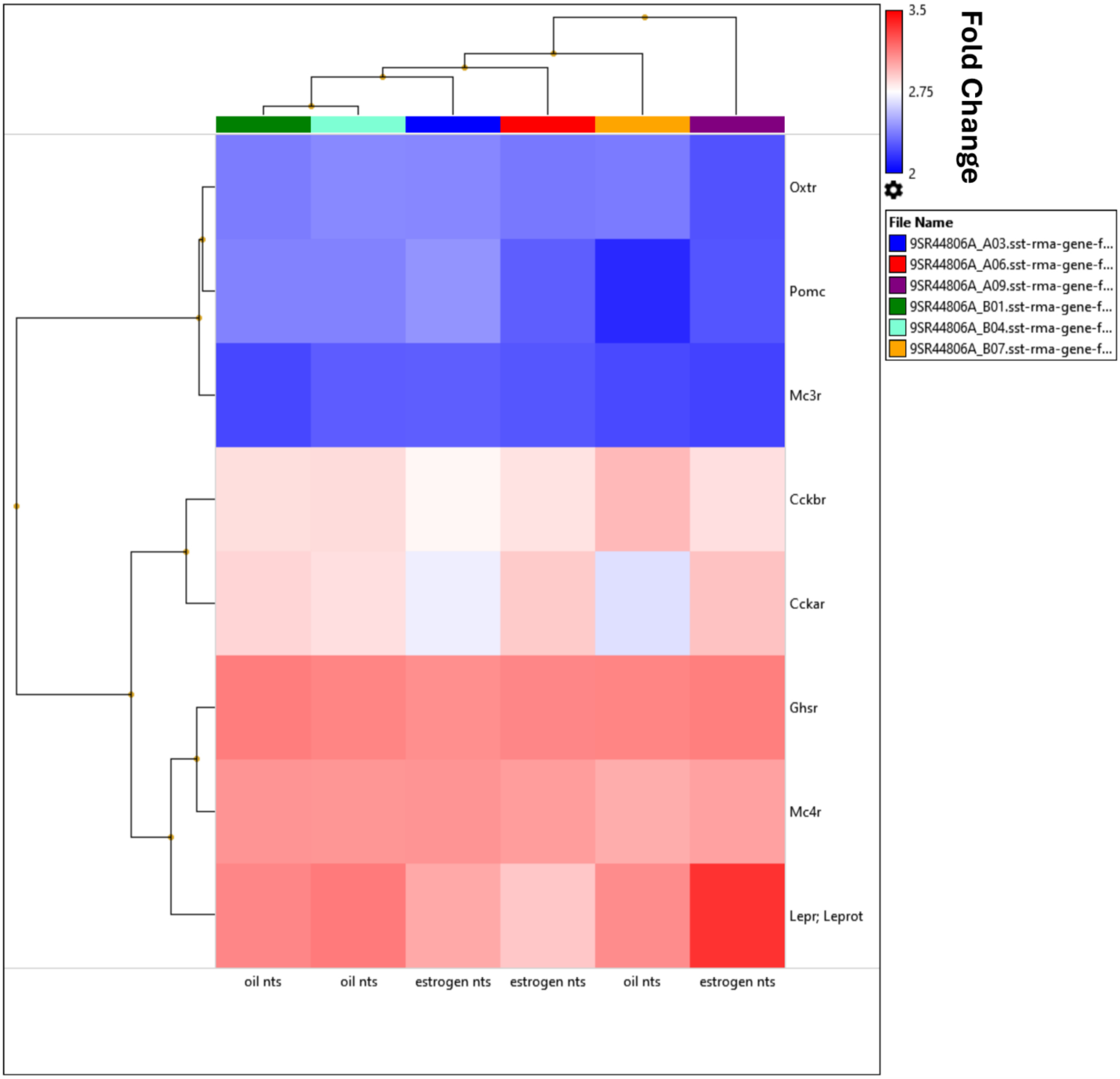

Supplement: Supplementary file 2 — Additional file 2. Heatmaps_Selected Genes. Heatmaps for genes of interest in the ARC, PVN, and NTS from EB- vs. Oil-treated samples. Heatmaps were generated using TAC to visualize the expression of selected genes of interest in the ARC (a), PVN (b), and NTS (c). Each column represents an individual sample; each row represents a single gene. Expression values are shown as log 2 transformed signal intensities. Red indicates the highest expression and blue indicates the lowest [file 13104_2026_7672_MOESM2_ESM.pdf]
