# Supplementary material for: An exploratory microarray analysis of estrogen-mediated gene expression in central pathways that control energy balance in female rats (Rattus norvegicus)
Source: BMC Res Notes. 2026 Jan 30;19:88. doi: 10.1186/s13104-026-07672-2 (PMC12930718; doi:10.1186/s13104-026-07672-2)

Additional File 3_Heatmaps_DEGs

Title: Differentially Expressed Genes (DEGs) in the ARC, PVN, and NTS from EB- vs. Oil-treated samples

Description of Data:

Heatmaps were generated using TAC to visualize DEGs in the :

- ARC (a)
- PVN (b)
- NTS (c)

Genes were selected based on unadjusted *p*-values ≤ 0.05 and absolute fold change > 2. With the exception of Asb15 in the NTS, no genes met significance thresholds after FDR correction, therefore these heatmaps are presented for exploratory purposes only.

Each column represents an individual sample, and each row represents a single gene. Expression values are shown as log 2 transformed signal intensities. Red indicates the highest expression, and blue indicates the lowest.

3a ARC – DEGs in EB- vs. Oil-treated samples

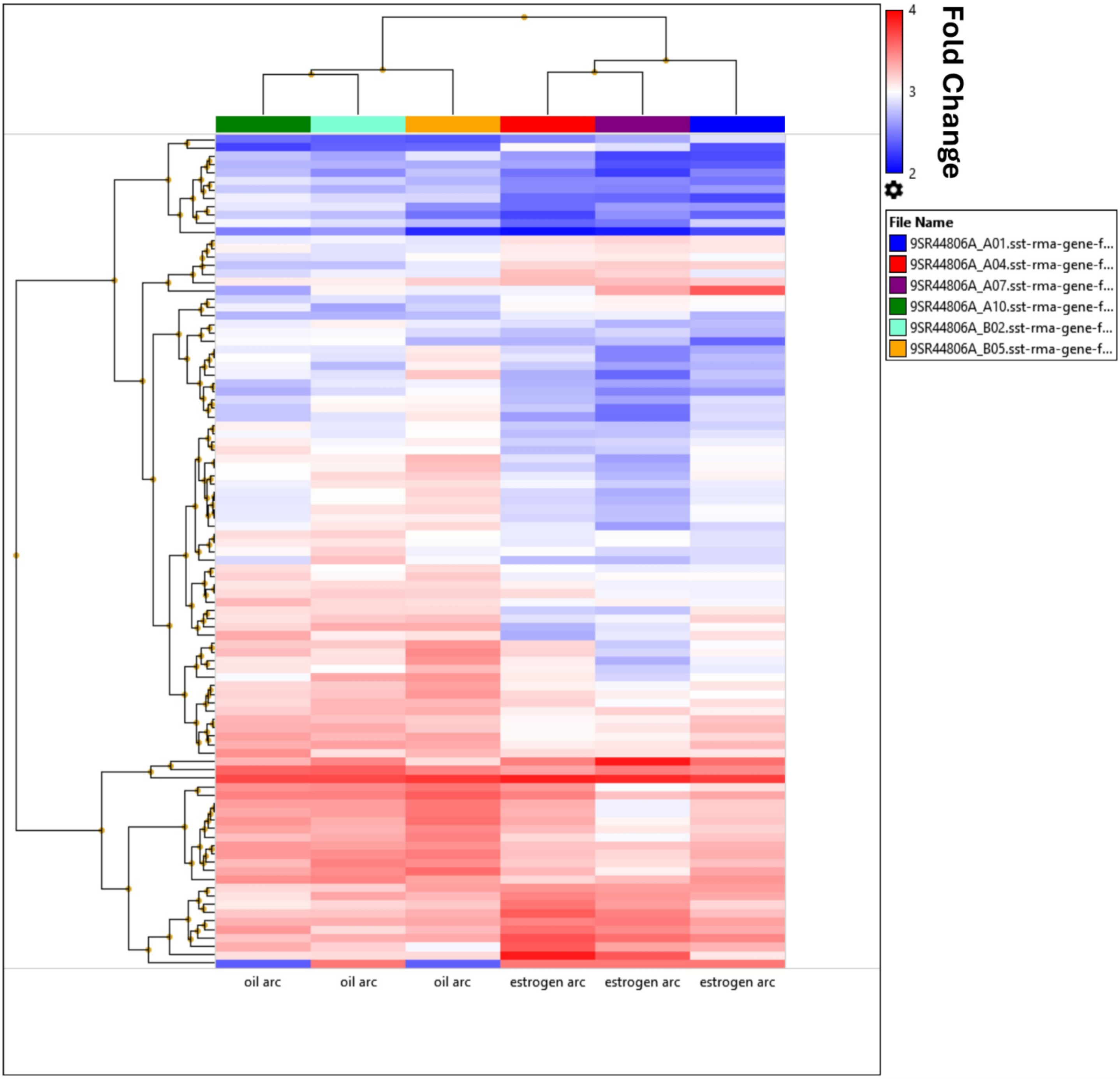

3b PVN – DEGs in EB- vs. Oil-treated samples

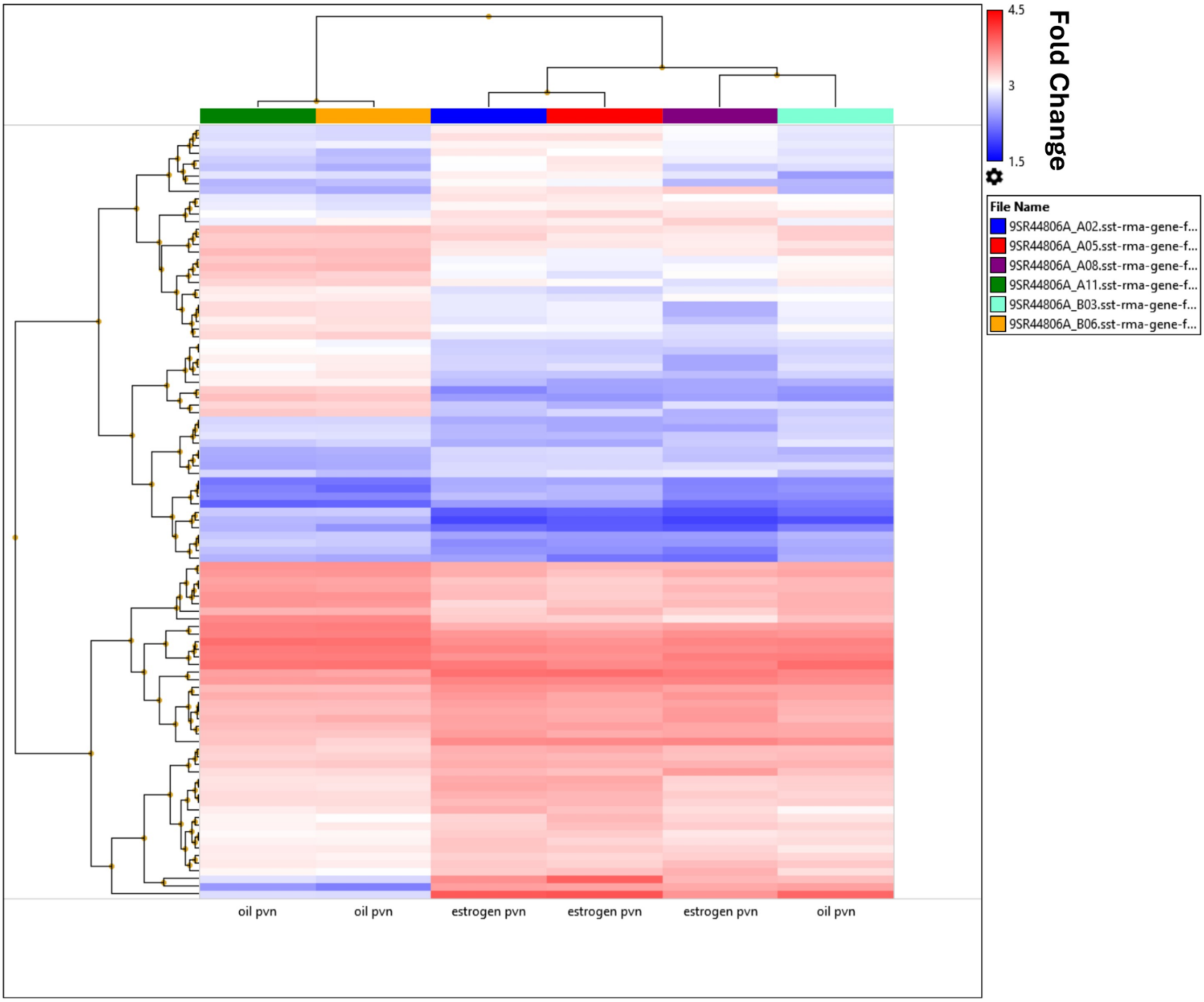

3c NTS - DEGs in EB- vs. Oil-treated samples

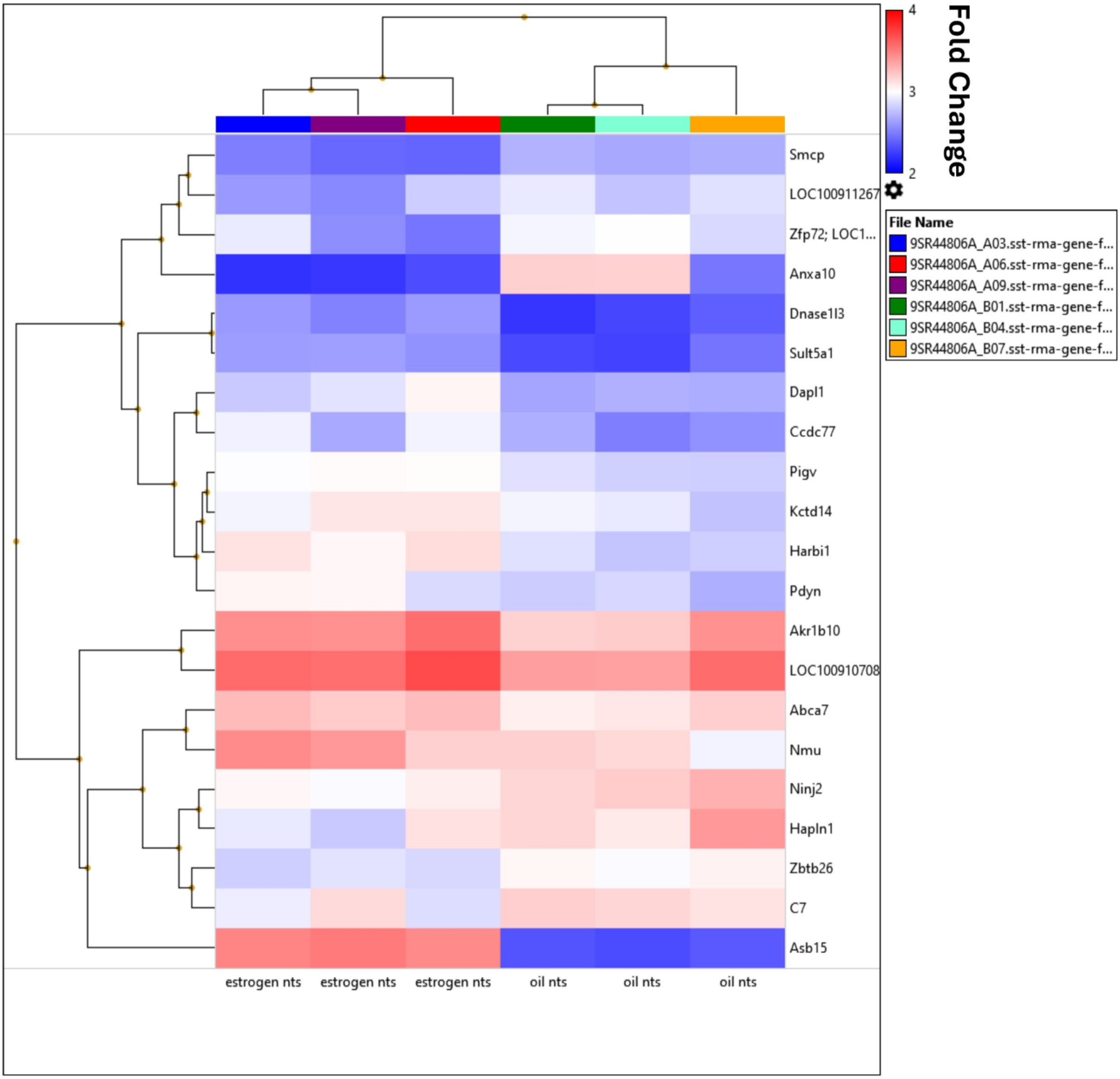

Supplement: Supplementary file 3 — Additional file 3. Heatmaps_DEGs. Differentially expressed genes (DEGs) in the ARC, PVN, and NTS from EB-vs. Oil-treated samples. Heatmaps were generated using TAC to visualize DEGs in the ARC (a), PVN (b), NTS (c). Genes were selected based on unadjusted p-values ≤ 0.05 and absolute fold change > 2. With the exception of Asb15 in the NTS, no genes met significance thresholds after FDR correction, therefore these heatmaps are presented for exploratory purposes only. Each column represents an individual sample, and each row represents a single gene. Expression values are shown as log 2 transformed signal intensities. Red indicates the highest expression, and blue indicates the lowest. [file 13104_2026_7672_MOESM3_ESM.pdf]
